# Application of hand-held mobility spectrometers as sensors in manufacturing industries

**DOI:** 10.1155/S1463924698000017

**Published:** 1998

**Authors:** Graeme Allinson

**Affiliations:** School of Aquatic Science and Natural Resources Management Deakin University PO Box 423 Warrnambol Victoria 3280 Australia

## Abstract

Ion mobility spectrometers (IMS) are small, lightweight, extremely robust devices with low power requirements, no moving parts, no absolute requirement for gases or vacuums, that can be operated at ambient temperatures and pressures, and yet are capable of measuring vapour phase concentrations of organic chemicals at very low levels (sub-μg/l). IMS are capable of analysing complex mixtures and producing a simple spectral output. Volatile components produce measurable negative and positive product ions in the spectrometer through chemical ionization. The spectra produced are essentially the vapour phase fingerprints of the target molecules/mixture. Quantitative data can be obtained provided instrument response is within the linear dynamic range of these instruments, but most practical applications of IMS have used the technology in a qualitative manner in situations which require just an above/below threshold or positive/ negative response.

In the manufacturing industry there are many examples where the aroma/odour of raw materials has safety or product quality implications. IMS was not developed to replace traditional methods of analysis, e.g. GC/MS or sensory panels, but rather to provide a rapid, qualitative response complementary to more established methods. This paper reports on the use of a hand-held ion mobility spectrometer to characterize the vapours produced by volatile organic compounds,fresh herbs and retail spice mixtures at ambient temperature and pressure. The results show that by monitoring in both ion acquisition modes, ion mobility spectrometers are capable of discriminating between a wide range of products.


Journal of Automatic Chemistry, Vol. 20, No. 1 (January–February 1998) pp. 1–7

### Application of hand-held mobility spectrometers as sensors in manufacturing industries

**Graeme Allinson**
*School of Aquatic Science and Natural Resources Management, Deakin University, PO Box 423, Warrnambol, Victoria 3280, Australia*

*Ion mobility spectrometers (IMS) are small, lightweight, extremely robust devices with low power requirements, no moving parts, no absolute requirement for gases or vacuums, that can be operated at ambient temperatures and pressures, and yet are capable of measuring vapour phase concentrations of organic chemicals at very low levels (sub-µg/l). IMS are capable of analysing complex mixtures and producing a simple spectral output. Volatile components produce measurable negative and positive product ions in the spectrometer through chemical ionization. The spectra produced are essentially the vapour phase fingerprints of the target molecules/mixture. Quantitative data can be obtained provided instrument response is within the linear dynamic range of these instruments, but most practical applications of IMS have used the technology in a qualitative manner in situations which require just an above/below threshold or positive/negative response.*

*In the manufacturing industry there are many examples where the aroma/odour of raw materials has safety or product quality implications. IMS was not developed to replace traditional methods of analysis, e.g. GC/MS or sensory panels, but rather to provide a rapid, qualitative response complementary to more established methods. This paper reports on the use of a hand-held ion mobility spectrometer to characterize the vapours produced by volatile organic compounds, fresh herbs and retail spice mixtures at ambient temperature and pressure. The results show that by monitoring in both ion acquisition modes, ion mobility spectrometers are capable of discriminating between a wide range of products.*

### Introduction

Human beings can detect odours or suffer adverse health effects when the volatile compounds causing them are present in the atmosphere at very low levels (sub-µg/l). Any nonbiological sensor proposed as a replacement for expert panels, as an adjunct on production lines or an alarm monitor must be able to respond to odours, or at least the different chemical functional groups found on the volatile molecules producing the odour, at similar concentrations to humans. An instrument that has the potential for the rapid screening of vapours without extensive sample pretreatment is the ion mobility spectrometer (IMS). Ion mobility spectrometers are small, lightweight devices with no moving parts, no absolute requirement for bottled gases or vacuums, that can be operated at ambient temperatures and pressures using a 6 V power supply and yet are capable of measuring extremely rapidly vapour phase concentrations of organic chemicals at very low levels (sub-µg/l) [1]. Mobility spectrometry has been known as an analytical technique since the early 1970s [2, 3]. However, the presence of IMS instrumentation in only a few laboratories has limited the the number and diversity of applications. Most applications have had military or forensic origins, including the detection of chemical warfare agents [4], explosives [5], narcotics [6] and tear gases [7]. Other reported uses of IMS appear to have been, to a large extent, a spin-off from military chemical warfare agent detection and monitoring programs. These include the detection of nicotine [8], airborne organic and inorganic vapours [9, 10], polycyclic aromatic hydrocarbons [11], the identification of polymers [12], ammonia in aqueous samples [13], and benzene in ambient air [14]. Applications of IMS in manufacturing have been limited and include the detection of coliforms in processed foods [15, 16], outgassings of polymers used in the semiconductor industry [17], detection of northern red oak wetwood [18], to measure ethanol in beer and yeast fermentation [19], determining the freshness of fish [20], or as a GC detector in the quantification of mammalian lignans in biological fluids [21]. The wide range of compounds detectable by IMS was graphically illustrated in three review lists published by manufacturers of this instrumentation [22–24], and include acid and stack gases, hydrazines, amines, isocyanates, chlorinated solvents, DMSO, methyl salicate, halothane, enflurane, isoflurane, diethyl ether, acetone and methyl ethyl ketone.

The type of sensory information required determines whether a single sensor or an array of sensors is needed to achieve a unique spectral fingerprint from a raw material/product which may be emitting a complex vapour. A detailed knowledge of the species causing the spectra is an absolute requirement only if quantitative information is required. This paper stresses the great potential of IMS when used in a qualitative or alarm response manner. In the study reported here, a hand-held IMS was used to characterize the vapours produced by nicotine, α-chloroacetophenone, malononitrile, and fresh herbs and spice mixtures at ambient temperature, and in both positive and negative ion acquisition modes. The results suggest that mobility spectrometers can be used qualitatively to characterize the presence of a target molecule, the composition of a mixture, and initiate an alarm response when conditions differ from a predetermined value.

0142-0453/98 $12·00 © 1998 Taylor & Francis Ltd

### Experimental

### *Materials*

Nicotine and malononitrile (Aldrich Chemical Co. Ltd, Poole, UK) were used without further purification; $\alpha$-chloroacetophenone (Lancaster Synthesis Ltd, Morecambe, UK) was recrystallized twice from hexane before use. Fresh herbs (basil, black peppercorns, cinnamon, cloves, coriander, dill, garlic, ginger, green cardomon, mint, nutmeg, oregano, parsley, rosemary, tarragon and thyme) and commercial spice mixtures (Cajun seasoning, cayenne pepper, garam masala, 'hot' chilli powder, 'hot' Mexican seasoning, 'hot' paprika, 'hot' Vindaloo curry powder, Madras curry powder, Thai 7 spice seasoning and Szechuan seasoning) were obtained from a local retail outlet.

### *IMS system*

A Graseby Ionics airborne vapour monitor (AVM) capable of operating in both the positive and negative ion acquisition modes. Spectra were accumulated using a Graseby analytical ASP board controlled by the proprietary WASP data acquisition software (Graseby Ionics, Bushey Road, Watford, UK).

### *General operation*

At the simplest level of operation, a stand-alone IMS [figure 1(*a*)] recycles a reagent gas through an internal filter system. Some ionization of the reagent gas takes place in the spectrometer ionization chamber. The reagent ions pass through the drift region of the device under the influence of an electric field, eventually being detected by the detector as an ion current. This gives rise to a signal which may be expressed as a single spectral peak known as the reactant ion peak [RIP, figure 1(*b*)].

The introduction of a volatile component into the spectrometer produces additional negative and positive product ions through reactant ion–sample vapour molecule interactions. These also pass through the drift region under the influence of the electric field and, encountering the counter flow of drift gas, are separated by both charge and size. These ions give rise to spectral peaks at characteristic drift times relative to that of the RIP [figure 1(*c*)]. This chemical ionization of sample vapours produces spectra that are essentially the vapour phase fingerprints of the target molecules. Quantitative data can be obtained provided instrument response is within its linear dynamic range. Unfortunately, the linear dynamic range of these instruments is small—typically only two orders of magnitude—so most practical applications of IMS have used the technology in a qualitative manner in situations which require just an Above/Below Threshold or Positive/Negative response [1, 2].

### *General method*

The samples were placed at the bottom of a 500 ml round-bottomed flask. The stoppered flasks were left at ambient temperature (20–25°C) for 10 min. Thereafter, the atmosphere inside the flasks was sampled by introducing the inlet nozzle of the IMS into the neck of the flask and acquiring data over a period of approximately 30 s. Repetition of this process allowed the selection of spectra typical of the steady-state response produced by the instrument.

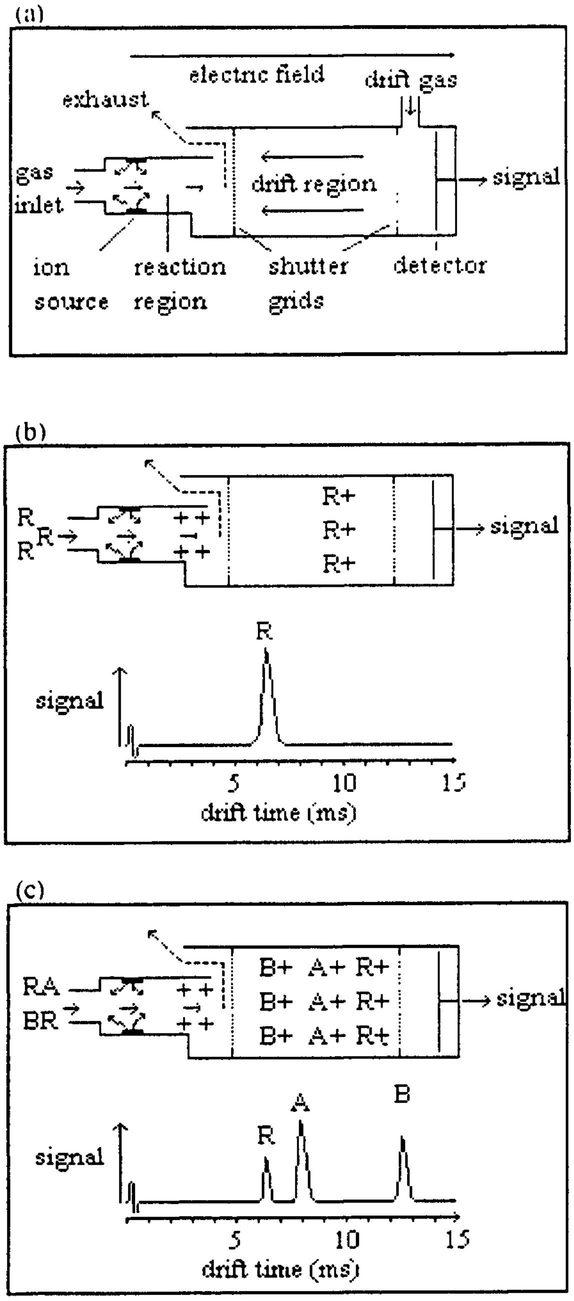

*Figure 1. General principles of IMS operation.*

### Results and discussion

The type of product and the production process will dictate the suitability and mode of operation of any sensor. A wide range of compounds may be detected by IMS, for example acid and stack gases, hydrazines, amines, isocyanates, chlorinated solvents, DMSO, methyl salicate, halothane, enflurane, isoflurane, diethyl ether, acetone and methyl ethyl ketone [22–24]. Ion mobility spectrometry has possibly its greatest potential

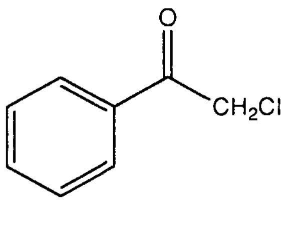

I

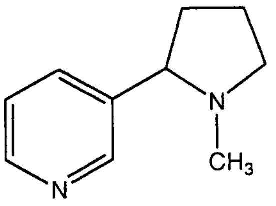

II

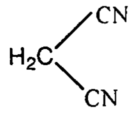

III

*Figure 2. Chemical structures of target molecules.*

in the rapid monitoring of products containing a single volatile component of high proton affinity/high electronegativity, for example, α-chloroacetophenone [figure 2, (**I**), nicotine (**II**), or malononitrile (**III**)].

A hand-held IMS, such as the Graseby Ionics AVM, is primarily designed to sample ambient air without any sample vapour preseparation. The method used to prepare and analyse the samples was simple but effective. When sampling the atmosphere inside clean flasks no depression of the RIP was observed. Spectral reproducibility was evaluated using two α-chloroacetophenone standards—5 μg and 37·5 μg. Typical positive and negative ion acquisition mode spectra for this compound are shown in figure 3. Samples were analysed in both ion acquisition modes in batches over four days. The steady-state reproducibility was found to be peak area and background noise dependent. Put simply, the greater the peak area the smaller the coefficient of variance (3–17%). The day-to-day variation in positive and negative mode signal intensities was to some extent dependent on atmospheric temperature, pressure and humidity, but was not significantly different from the single-flask variance. Ultimately, instrument sensitivity was most affected by repeatedly switching between positive and negative ion acquisition modes during sampling. The spectra discussed hereafter were acquired under such conditions and, while not showing the AVM at its most

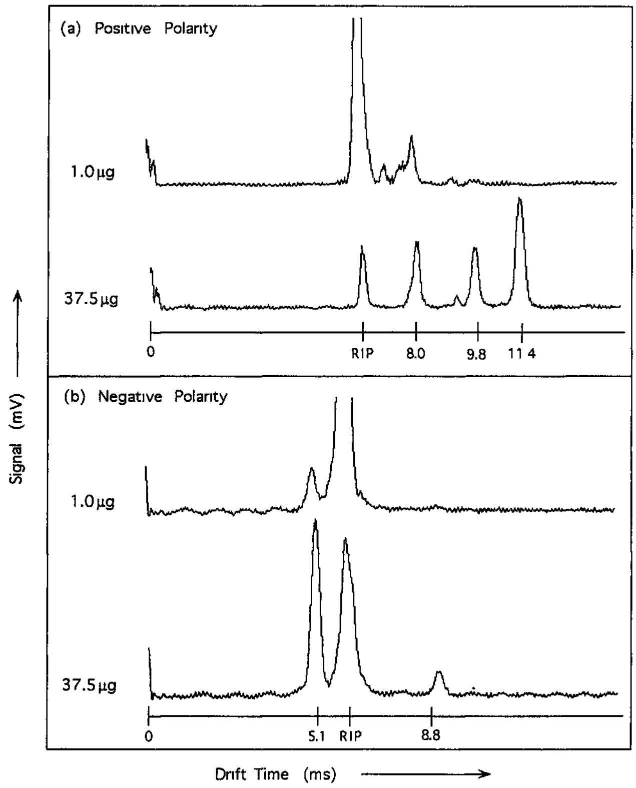

*Figure 3. Typical positive and negative ion acquisition mode spectra obtained from 1 and 37·5 μg of α-chloroacetophenone at ambient temperature.*

sensitive, represent the type of dual mode sampling strategy that practical use of this instrument may require.

A significant potential application for mobility spectrometers is that of a highly sensitive, static atmospheric alarm monitor for noxious vapours. In this study, α-chloroacetophenone (**I**), nicotine (**II**) and malononitrile (**III**) were used to illustrate the potential of IMS to respond to different functionality, for example, ketone groups (in **I**), pyridyl nitrogen (in **II**), and nitrile groups (in **III**).

Aerosol spray cans containing lachrymatory chemicals such as α-chloroacetophenone (the active ingredient of CN tear gas) have increased in popularity in recent years as both personal protection devices and as antipersonnel devices used during the commission of crimes. Acute exposure to the active ingredients of tear gas produces severe irritation of the skin, eyes and mucous membranes, severe couching, nausea and vomiting. Severe exposure has led to fatalities [25]. At low residue levels and hence low vapour concentrations, the positive polarity IMS response to α-chloroacetophenone consists only of the peak at 8·0 ms [$MH^+$; figure 3(*a*)]. As the vapour concentration increased, the peaks at 9·8 and 11·4 ms emerged. This latter peak, possibly ($MH^+L_n$, $M_2H^+$ or even $M_2H^+L_n$) finally dominated the spectrum. This compound also produces negative polarity spectra. Those observed when sampling the atmosphere above 5 μg and 37·5 μg samples of α-chloroacetophenone at ambient temperature are shown in figure 3(*b*). At low residue levels and hence low vapour concentrations, only the peak at 5·1 ms was observed ($Cl^-$). As the vapour concentration increased the peak at 8·8 ($MCl^-$) emerged.

Nicotine (**II**) is a ubiquitous contaminant of workplace air. This compound is volatile with particularly highly proton affinitive functional groups. Nicotine does not produce a negative ion acquisition mode response, but is known to produce a strong positive polarity IMS response [8]. The positive ion acquisition mode (positive polarity) response to the vapours emitted by 12·5 and 250 µg of nicotine at ambient temperature is illustrated in figure 4. As with the positive polarity spectra produced by α-chloroacetophenone, one might conclude that a number of different compounds are causing the spectra. This is not the case. The spectra are indeed the result of a single, pure compound. The spectal peaks arise from a number of interrelated processes. The first stage in any IMS measurement is the ionization of the neutral sample molecules. In general, ionization is based upon electron or proton transfer events arising from gas phase collisions and subsequent chemical reaction of charged reagent ions and sample vapours. The major feature that distinguishes this method of ionization is the distribution of charge among sample molecules according to their gas phase proton or electron afffinities. For instance, in positive polarity charge can be distributed to virtually all compounds when the reagent ion is of low proton affinity, i.e. a hydrated proton. Theoretically, then the IMS responds to a large number of compounds. However, charge transfer is competitive. Charge transfer from the reactant ions can be to a single chemical, several chemicals or, indeed, a large number of compounds, and is governed primarily by the relative proton affinities and concentrations of the individual components of the mixture [1]. An added complication in interpreting mobility spectra is that for most organic compounds the instrument does not respond in a linear manner with increasing vapour concentration when using air as the reagent gas at ambient temperatures and pressures. Product ions are prone to forming cluster ions where a charged molecule is associated with a neutral compound. The large number of collisions, low ion energies and relatively high vapour densities in the spectrometer's reaction region [figure 1(*a*)] promote such ion–molecule associations [1]. In nicotine's case, at low residue levels and hence low

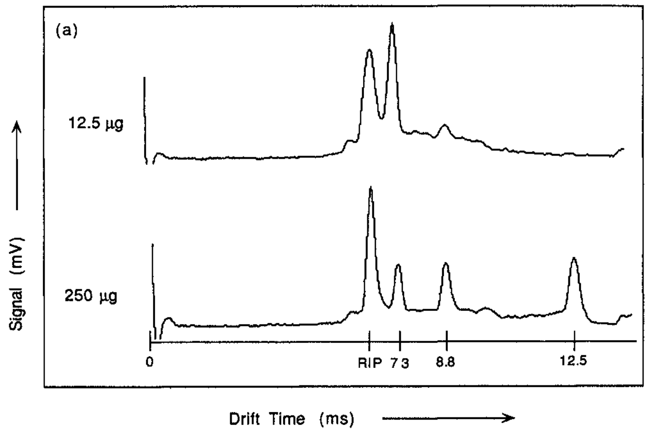

*Figure 4. Typical positive ion acquisition mode spectra obtained from 12·5 and 250 µg of nicotine at ambient temperature.*

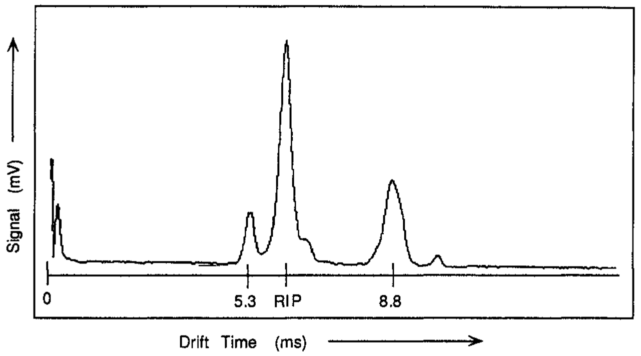

*Figure 5. Typical negative ion acquisition mode spectra obtained from 0·5 µg malononitrile at ambient temperature.*

vapour concentrations, only the peak at 7·3 ms is consistently observed. This peak is probably due to a monomeric molecular ion of the form ($MH^+$). As vapour concentrations increase, the peaks at 8·8 ms and 12·5 ms emerge sequentially. Eventually this latter peak, possibly a cluster ion of the form ($MH^+L_n$), a dimer ($M_2H^+$) or even a clustered dimer ($M_2H^+L_n$), dominates the spectrum. Detailed information on the exact nature of the ion generating IMS signals is not available directly from a hand-held spectrometer system. More detailed investigations using IMS/MS would be required to determine such information.

The typical negative ion acquisition mode spectra obtained when sampling the atmosphere above 25 µg of malononitrile at ambient temperature is shown in figure 5. The spectrum is relatively straightforward, consisting of a peak at 5·3 ms ($Cl^-$) and an albeit unusually broad peak at 8·9 ms (possibly due to $MCl^-$). It does, however, illustrate the extreme sensitivity of IMS technology towards nitrile groups.

The discussion so far has concentrated on spectra produced by a single, pure compound. But what of mixtures, or samples emitting a more complex mixture of volatile compounds, such as foodstuffs? Food flavour is important in defining product type and consumer preference. Human beings experience flavour as a result of a synergy between taste and smell. The sense of taste is caused by the interaction of nonvolatile compounds with biological sensors in the tongue and mouth, that of smell by reaction of volatile compounds with sensors in the nose. It is these latter volatile molecules that are responsible for odours—pleasant or unpleasant. The thousands of volatiles which make up odour may arise from the food itself or as a result of its processing—e.g. roasting and toasting generate typical aromas resulting from thiazoles, pyrazines and pyridines. Some odours are associated with freshness and wholesomeness, others with food being 'off' or tainted. These latter 'bad' aromas may arise as a result of decomposition or contamination—e.g. ammoniacal, musty/mouldy and putrid odours resulting from volatile amines, aromatics and thiocompounds. The volatile compounds associated with these aromas may also produce an IMS response in one, or both, ion acquisition modes depending on the nature of the chemical functionality of the molecules. Again, competitive charge transfer is important. The spectra observed will be governed

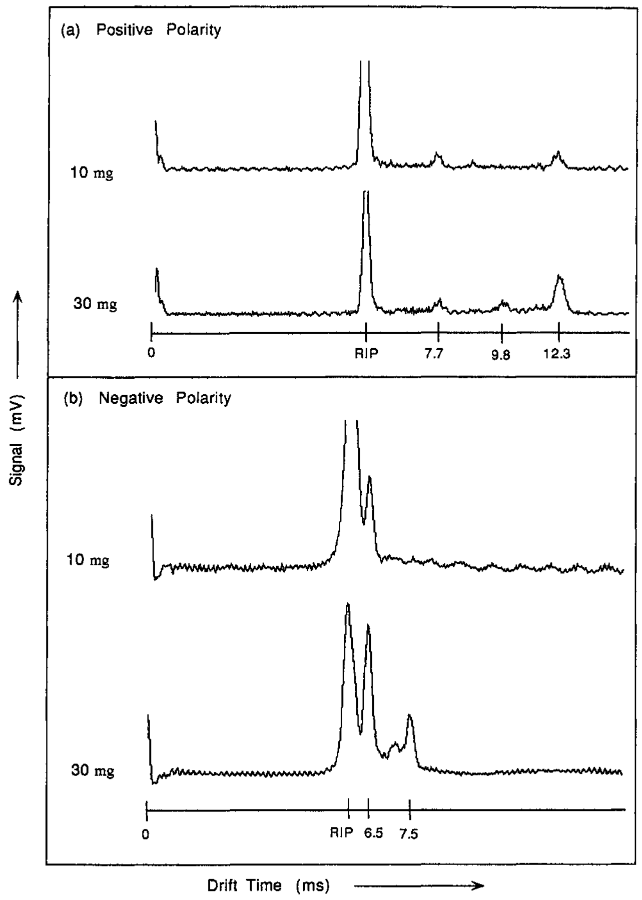

*Figure 6. Typical positive and negative ion acquisition mode spectra obtained from 10 mg of chilli powder at ambient temperature.*

primarily by both the concentration and relative proton/electron affinities of the individual components of the mixture [1]. Figure 6 shows the characteristic mobility spectra obtained when the atmosphere above 10 mg and 30 mg of chilli pepper are sampled sequentially in positive and negative ion acquisition modes at ambient temperature. The spectra may be enhanced by sampling increased quantities of chilli powder, or by raising the temperature of the sample. In an industrial setting, an IMS situated close to a heated production line may produce a significantly enhanced response, because of increased volatilization of target compounds, e.g. nicotine emitted by tobacco leaves emerging from a drier [26]. Figure 7 shows the typical positive and negative ion acquisition mode spectra produced by 10 mg of fresh mint, garlic, cinnamon and nutmeg. Although there is a great deal of similarity in the negative ion acquisition mode spectra, probably the result of the emission of similar volatile compounds, the positive polarity spectra are significantly different. Figure 8 shows the typical positive and negative ion acquisition mode spectra produced by 10 mg of some commercial spice mixtures. There is a great deal of similarity in both negative and positive ion acquisition mode spectra. Since these spice mixes contain essentially the same ingredients merely blended in different proportions, this similarity is probably the result of the emission of identical volatile compounds. Again, IMS/MS investigations are required to

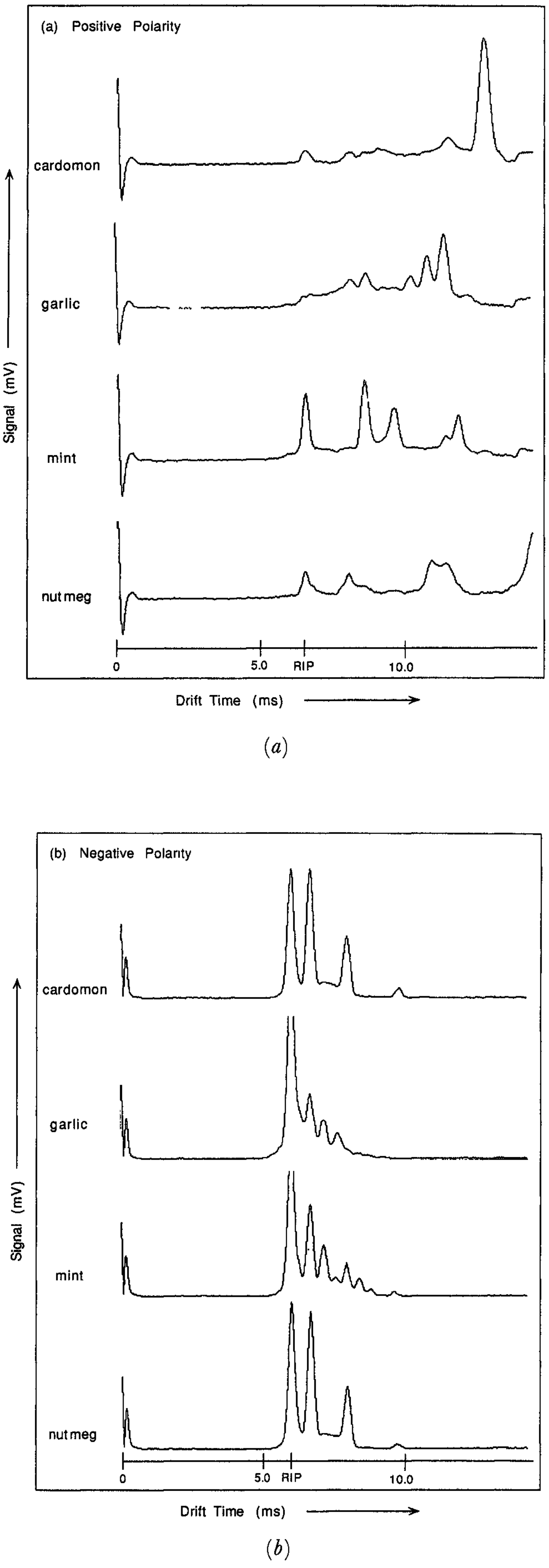

*Figure 7. Typical positive and negative ion acquisition mode spectra obtained from 10 mg of fresh herbs at ambient temperature.*

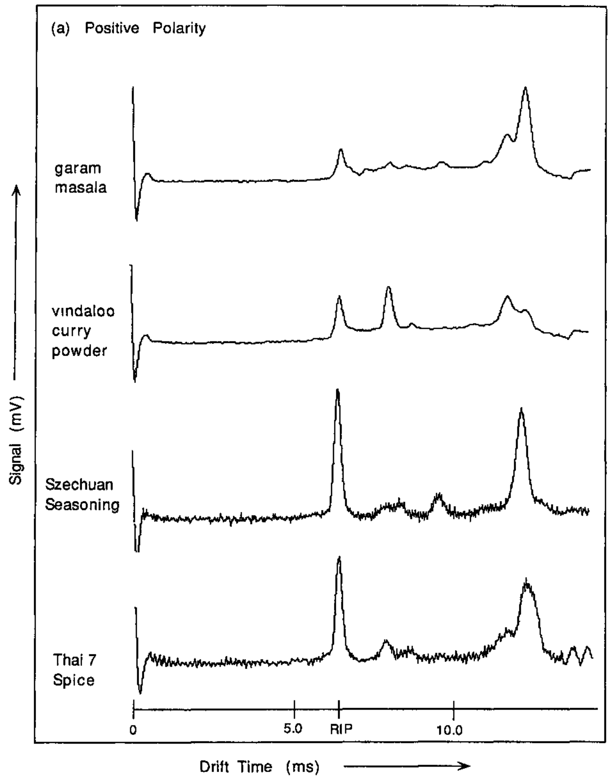

(*a*)

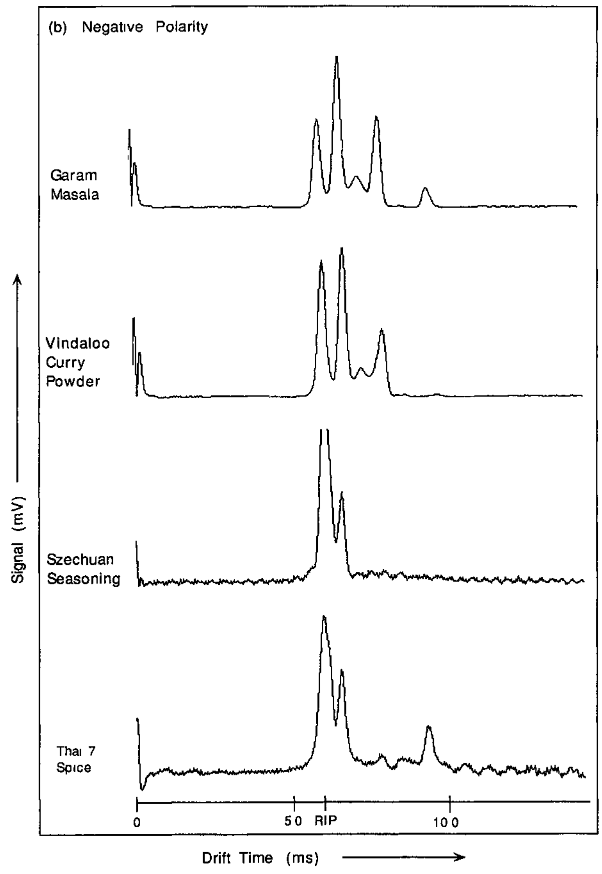

(*b*)

*Figure 8. Typical positive and negative ion acquisition mode spectra obtained from 10 mg of fresh herbs at ambient temperature.*

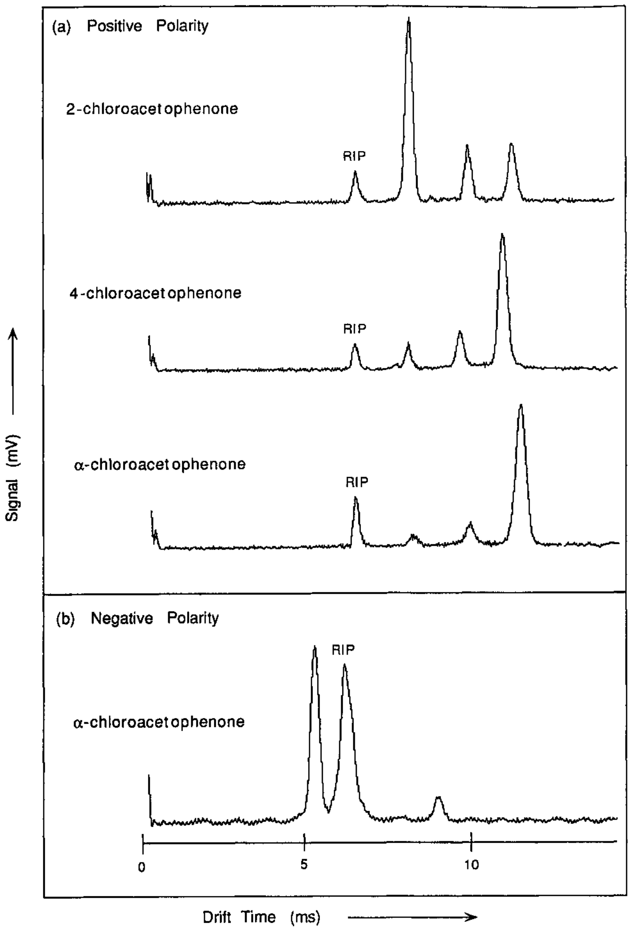

*Figure 9. Differentiation of mono-chloroacetophenones by sequential positive and negative ion mode IMS.*

interrogate the identity of the species causing the signals observed with the herbs and spices. However, these results do illustrate one of the major strengths of mobility spectrometers—the ability to obtain spectra in both ion acquisition modes sequentially. By analysing first in the negative ion mode, then in the positive, one adds an extra dimension to compound identification and it is possible to discriminate between closely related compounds, for example, between the three monochloroacetophenone compounds α-chloroacetophenone, 2-chloroacetophenone and 4-chloroacetophenone (figure 9), or between spice mixtures containing essentially the same ingredients blended in different proportions (figure 8).

### Neural networks

Once mobility spectra are available, neural network based pattern recognition algorithms can be written. Such powerful computer programs are required because although mobility spectrometers have excellent detection limits and near real-time operation, their applications have been limited due to difficulties associated with the analysis of their complex signatures—essentially the problem is not getting IMS spectra but in being able to identify a characteristic chemical fingerprint within a complicated spectrum [27]. The advent of artificial neural network algorithms—and two in particular, the error back propagation network [28] and the cascade correlation network [29]—have provided an opportunity to significantly enhance the capabilities of this instrument. Artificial neural networks are highly parallel adaptive systems capable of learning by example. They are especially effective in cases where signals are noisy, and in cases where the statistical distribution of data is such that conventional data analysis is not applicable, e.g. mobility spectra [30]. Neural network algorithms will undoubtedly be at the heart of any monitoring device based on IMS technology alone, i.e. without pre-separation of vapours before analysis.

### Conclusions

Mobility spectrometry has significant potential for use as an alarm monitor continuously sampling factory air for hazardous volatile organic compounds, or as a quality control sensor in cases where a product contains a single, volatile substance. Complex mixtures produce quite complex spectra which generally do not simplify on increasing the sampling temperature. This suggests that these spectra are produced by a mixture of compounds differing in volatility and proton/electron affinity rather than by a single compound prone to clustering and dimerization. However, by monitoring in both positive and negative ion acquisition modes, it is possible to obtain characteristic spectra. It should be possible to compare acquired spectra with those produced by standard, ideal, product mixtures and generate an action response when required. Spectral deconvolution and the use of neural network pattern recognition software will considerably enhance the speed of spectral identification.

### Acknowledgements

The author wishes to acknowledge the help and encouragement given within the Environmental Research Centre, Sheffield Hallam University by Michael Cooke and Cameron McLeod; by John Brokenshire and Michael Berryman of Graseby Ionics; and the financial support provided by the provision of a Visiting Research Fellowship by the Royal Society of London.

### References

1. Eiceman, G. A. and Karpas, Z., in *Ion Mobility Spectrometry* (CRC Press, Boca Raton, FL, 1994).
2. St. Lewis, H. and Hill, H. H. Jr., *CRC Critical Reviews Analytical Chemistry*, **21** (1990), 321.
3. Hill, H. H. Jr., Siems, W. F., St. Lewis, R. H. and McMinn, D. G., *Analytical Chemistry*, **62** (1990), 1201A.
4. Huang, D., Kolaitis, L. and Lubman, D. M., *Applied Spectroscopy*, **41** (1987), 1371.
5. Lawrence, H., *Analytical Chermistry*, **58** (1986), 1269.
6. Eiceman, G. A., *Critical Reviews Analytical Chemistry*, **22** (1991), 17.
7. Allinson, G. and McLeod, C. W., *Journal of Forensic Sciences*, **42** (1997), 311.
8. Keenan, F. and Cooke, M., *Analytical Proceedings Analytical Communications*, **31** (1994), 27.
9. Eiceman, G. A., Snyder, A. P. and Blyth, D. A., *International Journal of Environmental Analytical Chemistry*, **38** (1990), 415.
10. Eiceman, G. A., Leasure, C. S. and Vandiver, V. J., *Analytical Chemistry*, **58** (1986), 76.
11. Eiceman, G. A., Anderson, G. K., Danen, W. C., Ferris, M. J. and Tiece, J. J., *Analytical Letters*, **21** (1988), 539.
12. Simpson, M., Anderson, D. R., McLeod, C. W. and Cooke, M., *Analyst*, **118** (1993), 449.
13. Przybylko, A. R. M., Thomas, C. L. P., Anstice, P. J., Fielden, P. R., Brokenshire, J. and Irons, F., *Analytical Chimica Acta*, **311** (1995), 77.
14. Schecter, I., Schroder, H. and Kompa, K. L., *Analytical Chemistry*, **65** (1993), 1928.
15. Strachan, N. J. C. and Ogden, I. D., *Letters in Applied Microbiology*, **17** (1993), 228.
16. Strachan, N. J. C., Nicholson, F. J. and Ogden, I. D., *Analytica Chimica Acta*, **313** (1995), 63.
17. Budde, K. J., Holzapfel, W. J. and Beyer, M. M., *Journal of the Electrochemical Society*, **142** (1995), 888.
18. Petterson, R. C., Ward, J. C. and Lawrence, A. H., *Holzforschung*, **47** (1993), 513.
19. Kotiaho, T., Lauritsen, F. R., Degn, H. and Paakanen, H., *Analytica Chimica Acta*, **309** (1995), 317.
20. Snyder, A. P., Harden, C. S., Davis, D. M. and Shoff, D. B., Hand-portable gas chromatography-ion mobility spectrometer for the determination of the freshness of fish. Third International Workshop on Ion Mobility Spectrometry, Houston, TX (1994).
21. Atkinson, D. A., Hill, H. H., Jr. and Schultz, T. D., *Journal of Chromatography—Biomedical Applications*, **617** (1993), 173.
22. Roehl, J., *Applied Spectroscopy Reviews*, **26** (1991), 1.
23. Chemicals Detectable by ETG's IMS Product Line, GP/IMS and FP/IMS (Environmental Technologies group, Inc., Baltimore, MD 21284, USA).
24. Brochures, TDI/AVM Monitor, Airborne Vapour Monitor (Graseby Ionics, Watford, UK).
25. Thorburn, K. M., *Archives of Environmental Health*, **37** (1982), 182.
26. Allinson, G., unpublished results.
27. Saatchi, M. R. and Jervis, B. W., *Computing and Control Engineering Journal*, **2** (1991), 61.
28. Rumelhart, D. E., Hinton, G. E. and Williams, R. J., *Nature*, **323** (1986), 533.
29. Fahlman, S. E. and Lebiere, C., Carnegie Mellon University Report CMU-CS-90-100 (August 1991).
30. Harrington, P. de B., Zheng, P. and Davis, D. M., Quantitative analysis of volatile organic compounds using ion mobility spectra and cascade correlation neural networks, Third International Workshop on Ion Mobility Spectrometry, Houston, TX, 1994.

